# What Would Digital Early Intervention for Bipolar Disorder Look Like? Theoretical and Translational Considerations for Future Therapies

**DOI:** 10.3389/fpsyt.2019.00599

**Published:** 2019-08-23

**Authors:** Greg Murray

**Affiliations:** Centre for Mental Health, Swinburne University of Technology, Melbourne, VIC, Australia

**Keywords:** engagement, agency, transdiagnostic, online, smartphone, staging, co-design, resilience

## Abstract

There are growing calls for the development of early intervention/preventive interventions for young people identified to be at risk of bipolar disorder (BD), and digital delivery appears to be a strong candidate delivery method. To date, no such interventions exist, and the aim of this perspective paper is to advance the literature by reviewing theoretical issues related to early intervention in BD and introducing a framework for design of feasible, acceptable, and effective online psychosocial interventions for this population. It is concluded that, by adopting an appropriate transdiagnostic and humanistic framework, and recognizing emerging tenets of digital psychotherapy development, testable online interventions for young people at risk of BD are within reach.

Bipolar disorder (BD) is a serious mental illness affecting half a million Australians (1) and 2–3% of the world’s population (2). Prominent commentators are calling for the development of early intervention/preventive approaches to mitigate the significant morbidity and mortality associated with BD [e.g., Refs. (3–5)].^1^ Digital delivery platforms are receiving growing attention for established BD [e.g., Refs. (6–10)] but have not yet been applied in the early intervention domain. The aim of this paper is to take the next step toward online early intervention for BD by doing some prerequisite conceptual work. Firstly, existing data relevant to the potential of digital early intervention in BD are briefly summarized. Secondly, theoretical considerations for early intervention in BD are introduced, leading to three assertions about the optimal approach for those at elevated risk of BD. Finally, translational considerations are canvassed in a novel intervention design framework, the final stage of which is a co-design process with consumers.

## Digital Early Intervention for Bipolar Disorder: Some Relevant Data

To date, no digital early interventions have been developed for populations at risk of BD. However, triangulated findings from three cognate literatures suggest that digital platforms have potential as a modality for early psychological intervention in BD. Firstly, there is meta-analytic evidence that effective psychological treatments for young people *with, and at risk for, major depressive disorder* can be effectively and safely delivered digitally [see Refs. (11–13)]. In a recent review, Hollis and colleagues note that many questions remain in this nascent literature but conclude that digital health interventions hold “huge potential for widening access, increasing efficiency and improving healthcare outcomes” (14, p. 498). Secondly, a recent systematic review of early intervention for BD identified seven studies of *face-to-face psychological treatments* (15), with broadly positive findings for a range of outcomes. The existing literature has significant limitations, however: only two randomized controlled trials have been published, and the majority of studies have simply translated treatments for established BD, rather than testing bespoke interventions for early intervention in young adults (the emphasis here). Finally, online therapy as an adjunctive psychosocial treatment for *established BD* has been tested in a small number of trials, with generally positive findings on at least some outcome measures [e.g., Refs. (8, 9, 16, 17)]. Critically, our group has completed an international online trial of mindfulness-based therapy in late-stage BD with no reportable adverse events, providing some confidence about the safety of remote psychotherapy delivery in BD (18).

Arguments for the potential utility of digital delivery of mental health interventions include evidence for comparable effect sizes to face-to-face for many conditions, cost, and access (6, 19). Access considerations are particularly compelling in BD because less than half of people on the BD spectrum worldwide currently receive treatment of any kind (2). In combination with the empirical literature reviewed here, then, it can be concluded that there are strong *a priori* grounds for developing and testing digital early interventions for BD.

## Theoretical Context of Early Intervention in Bipolar Disorder

### Early Intervention and Stage-Tailoring

The rationale for early intervention in mental health is well accepted. As reviewed by Arango and colleagues (20), for example, existing research supports two major conclusions: there is increasing evidence for the benefits of universal and selective preventive interventions; and interventions targeting subthreshold presentations (indicated prevention) have potential to improve trajectories. In the domain of BD specifically, Vieta and colleagues recently proposed that early phases of the disorder may be more responsive to treatment and require less aggressive intervention, that there is an “at-risk” mental state for BD that can be used for indicated prevention, and that specific biological, environmental, and dimensional risk factors may be modifiable during this critical window (5).

The call for early intervention in BD overlaps conceptually with interest in stage-tailoring of treatments for BD. Several (largely compatible) clinical staging models have been described to capture the key features of BD within putative stages. These refer to an initial asymptomatic at-risk stage, followed by a stage characterized by nonspecific symptoms, and then a stage with more specific mood disorder-related, but subsyndromal symptoms. A syndromal stage, usually referred to as clinical stage 2, then follows, wherein mood episodes meet recognized diagnostic criteria and functional impacts begin to emerge followed by stage 3, where a repeated pattern of recurrences and relapses is common. The final or end stage (stage 4) is characterized by chronicity manifested by treatment refractoriness and progressively more severe functional impacts. Berk and colleagues (21) highlight the role of accumulating mood episodes and associated functional impairments. A related model proposed by Kapczinski and colleagues (22) prioritizes interepisodic functional and cognitive decrements as BD progresses.

Like the push for early intervention, enthusiasm for the staging approach in BD must be tempered by limited understanding of BD trajectories [see Ref. (23)]. Indeed, the notion that BD can be understood as a staged disorder is contentious [see Refs. (24, 25)]. Concerns about the staging heuristic include the potential for unproductive medication use [(26); see also Ref. (27)] and promulgation of the potentially demoralizing neuroprogression hypothesis [see Ref. (28)].^2^ Such concerns are particularly relevant to those “stages” falling early in the life course, given our incomplete understanding of the long-term effects of mood stabilizers on the developing brain [see Ref. (29)] and the potential to iatrogenically reinforce self-stigma and passivity (30–32).

A consequence of our imperfect developmental understanding of BD is lack of consensus on the best target population(s) for early intervention (33). Many studies to date have included family history as a risk factor (15), but the positive and negative predictive value of this criterion is limited, and so samples have often been “clinically enriched” by the presence of symptoms. Some research has focused on young people who are already presenting with hypo/manic symptomatology [e.g., Ref. (34)] or a less severe BD diagnosis (35). Research is ongoing to determine which clinical, social, and environmental factors may be associated with the development of BD for those at high familial risk [e.g., Ref. (36)]. The question of to whom should early intervention be offered (and consequently the specific targets of such intervention) has also been influenced by emerging transdiagnostic approaches (discussion later).

### Three Principles of Early Intervention: Minimize Harm, Attend to Transdiagnostic and Diagnostic Concerns, and Embrace Teleology

A tension therefore exists between two face-valid propositions about early intervention in BD—prompt attention to early signs could improve clinical outcomes, but without solid biopsychosocial understandings of disorder trajectories and treatment impacts, we are in danger of causing harm. I believe we can progress by acknowledging three principles of early intervention in BD.

First and foremost, early intervention attempts must attend to harm/benefit ratios. Given their more benign side-effect profiles, there is a consensus that any early intervention attempts should therefore privilege psychotherapies over pharmacotherapies [e.g., Ref. (37)]. Pragmatically, it has also been noted that young people are reticent to take medications for even diagnosed mental disorders, and clinicians are reticent to prescribe them (3). On the other hand, there is evidence that delay in instantiating first-line pharmacological treatments for BD is associated with negative outcomes (38). Taken together, these arguments suggest that interventions with stepped/sequential components may be optimal, with *adjunctive medication treatment* reserved for those who deteriorate or experience a diagnosable BD episode [for an example of one trial based around these principles, see Ref. (39)]. The focus of the present paper is the first, psychosocial step in this approach.

Secondly, it is useful to recognize a spectrum of specificity of problems/symptoms in populations at risk for BD, ranging for example, from nonspecific anxiety, through sleep/circadian problems, to relatively specific subsyndromal hypomania. In this vein, McGorry and colleagues argue that prevention/early intervention efforts must recognize pervasive pluripotence in psychopathology and so be organized around a broad range of inputs and outputs [e.g., Ref. (33)]. According to the Clinical High At Risk Mental State (CHARMS) (33) paradigm, inputs requiring attention are both disorder specific (family history of diagnosis, subsyndromal states, etc.) and transdiagnostic (e.g., functional decline). Similarly, outputs warranting attention should cover a range of target syndromes and problems. In translation, this paradigm would involve identifying distressed and help-seeking young people and targeting their presenting symptomatology (rather than any particular syndrome or proto-syndrome). The term “resilience” does useful work here (40, 41)—such transdiagnostic interventions are designed to address current issues and concomitantly build resilience against a range of negative health outcomes, including onset of frank BD.^3^

Finally, it is critical to expand our thinking beyond a medical paradigm, to explicitly recognize young people as motivated agents. The positive psychology [e.g., Ref. (42)] and recovery paradigms [e.g., Ref. (43)] remind us that a medical focus is only one side of the mental health coin. Complementary priorities are more explicitly teleological [explaining behavior by the outcomes it is intended to achieve, see Ref. (44)] and humanistic [prioritizing positive motivations and agency rather than abnormality and illness, see Ref. (45)] and, as such, will be particularly relevant in engaging young people with early intervention. Developmental psychopathology reminds us that young people at risk of BD are in a particular developmental window, working to optimize their well-being through completion of common developmental tasks [see, e.g., Refs. (46, 47)]. These theoretical observations have a very pragmatic implication—overlooking young people’s subjective quality of life (QoL) and meaning-making motivations will threaten engagement with any intervention we offer (46, 48, 49). Beyond the aim of improved resilience, then, early intervention can and should support young people building richness into their lives.

In sum, three principles can help navigate tensions between threats and opportunities in early intervention for BD. As we will see later, the various therapeutic targets implied by these principles can be addressed by a hybrid digital intervention drawing from existing evidence-informed psychosocial interventions.

## Considerations in Developing an Online Intervention for Young People at Risk of BD

**Figure 1:**
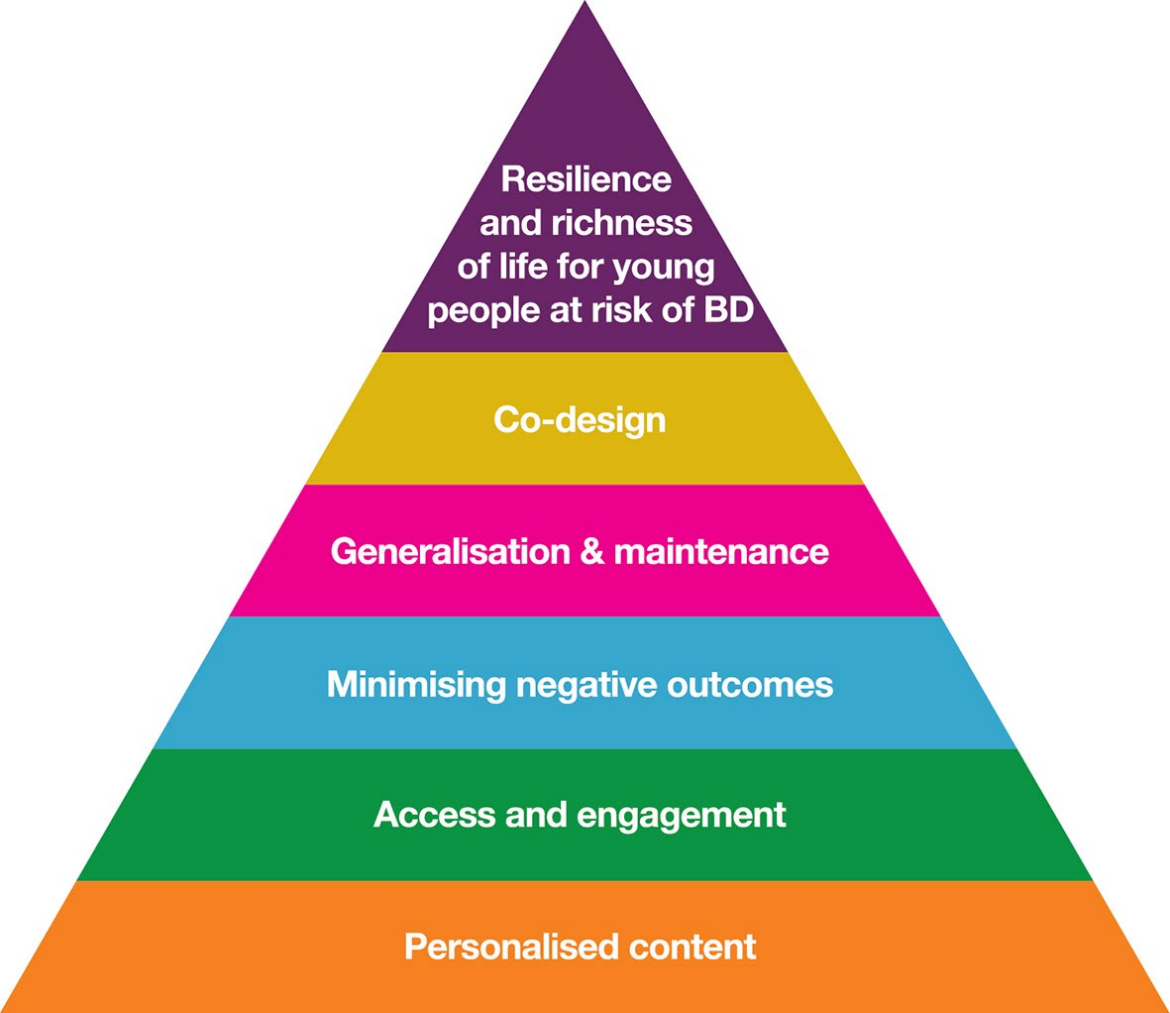
Intervention design framework for feasible, acceptable, and effective online intervention for young people at risk of BD.

There is clearly room for innovation in the space of digital early psychosocial intervention for BD. To support the translation of these principles, this section introduces an intervention design framework for future online interventions (**Figure 1**). It was noted earlier that a variety of subpopulations could be targeted as at risk for BD: To simplify the present exposition, “at risk” is defined as family history of BD, plus the presence of distress (with or without help-seeking behavior).^4^

**Figure 2:**
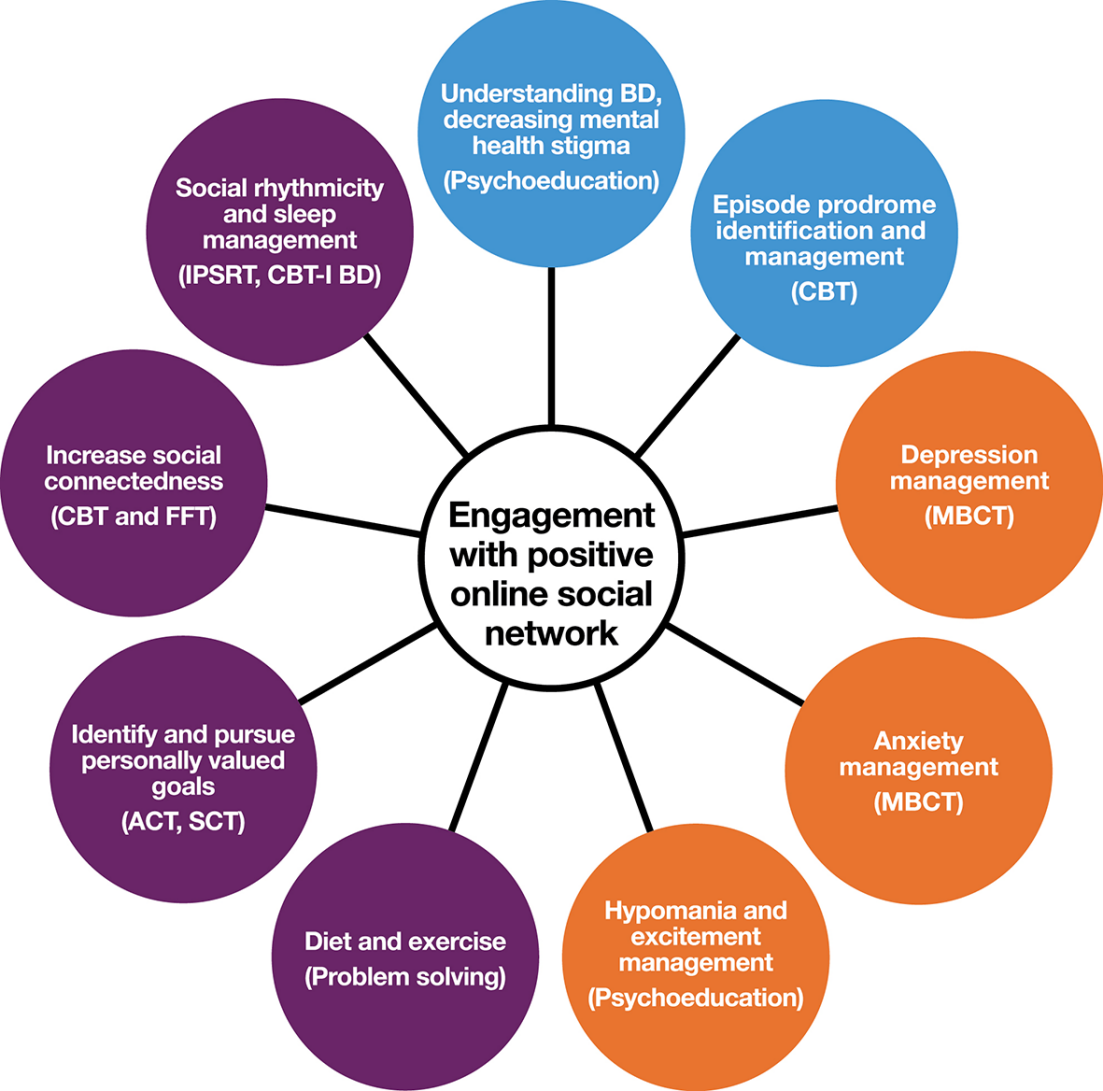
Possible elements of therapeutic content for online early intervention for young people at risk of BD. Blue (BD-specific) and purple (strengths-based targets) are core modules; Orange are optional modules based on young person’s current concerns (derived from empirical literature on prevalent problems in high-risk populations, or symptoms elevating risk). CBT, cognitive behavioral therapy; IPSRT, interpersonal and social rhythm therapy; MBCT, mindfulness-based cognitive therapy; ACT, acceptance and commitment therapy; SCT, self-compassion therapy; FFT, family-focused therapy; CBT-I BD, cognitive behavioral therapy for insomnia, modified for BD.

Following best practice, the design framework identifies specific, malleable causal factors (see **Figure 2**), a change mechanism (individual therapies with a recovery focus), and mode of delivery (email-supported web-delivery with social network components). The framework is evidence-informed, insomuch as it synthesises existing BD research into novel adjunctive psychotherapies (6, 28, 52, 53), technological delivery (18, 54), stage-tailoring (9), QoL outcomes (55–57), strengths (49, 58–60), co-design, and community-based participatory research (61). Note that the final level in the framework of **Figure 1** is co-design with consumers: the aim here is not to present a completed intervention, but to present a framework that can form the foundation of a future co-design process to develop such an intervention.^5^

### Personalised Content

The theoretical review presented above suggests that early intervention for young people at risk of BD should have hybrid targets including symptoms and syndromes linked specifically to BD, common nonspecific distress and problems of stage 1 BD, and also positive goals of QoL, and personally meaningful developmental goals. Summarized in **Figure 2** are approaches to achieving these diverse ends using modularized web-based delivery of evidence-based therapy components.

A critical target of early intervention attempts is prevention of hypomanic and manic episodes.^6^ Evidence-based therapies for established BD include development of knowledge and skills around personal relapse triggers and responding early to signs of impending relapse into a mood episode (62, 63). In early intervention, this “relapse prevention training” could be repurposed as “hypo/mania onset prevention training” through an interactive psychoeducation online module. Web delivery can also facilitate daily mood monitoring [e.g., *via* automatic generation of mood charts, see for example, Ref. (18)]. An exciting potential addition to monitoring self-reported mood is the integration of passive objective monitoring of activity *via* actigraphy (64–66), a technology that will likely be ready for clinical use in the near future. Key to objective monitoring as a clinical tool will be consideration of the human–technology interface: what type of information, in what form, and with what training, is most likely to encourage productive action?

Just as the epidemiology of staging in BD is unclear, the transdiagnostic symptoms and problems likely to be prevalent in this stage 1 population are not well characterized and differ depending on the operationalization of “at risk.” Possible transdiagnostic modules would include psychoeducation related to sleep, drugs and alcohol, physical activity and diet, and mental health stigma. Following the CHARMS approach, we note that this population of young people is heterogeneous, and engagement and efficacy demand the inclusion of optional online modules based on the young person’s experience (orange circles in **Figure 1**). Existing literature suggests that emotion regulation, anxiety, irritability, subsyndromal hypomanic, depression, and emotion regulation are likely to warrant attention as optional foci of work.

While diagnostic and transdiagnostic content can be addressed didactically through psychoeducation and cognitive-behavioral therapy, strengths-based content could be addressed through online versions of “third wave psychotherapies” (28, 67), with mindfulness and self-compassion strategies having untapped potential for improving QoL in BD (28). The values-based action principles from acceptance and commitment therapy (ACT) (68, 69) would be face-valid for encouraging young people to progress through developmental tasks [some of this content can be directed toward the likely strengths in this population, including creativity and a romantic aesthetic, see Ref. (49)].

### Access and Engagement

The great strength, of course, of online intervention is access: the web provides economical access to tailored psychological interventions and can overcome many barriers to accessing psychological assistance for BD, including cost, time, and trust in professionals (6). Online therapies are acceptable to people with established BD (70, 71), and the strengths of digital delivery are particularly pronounced in young adult populations (72).

Contemporary online interventions maximize engagement *via* best practice persuasive system design [see Refs. (54, 73, 74)], including: 1) dialogue support (praise from coach, email reminders, etc.), 2) social support (e.g., moderated forums), and 3) primary task support (modularization of content, personalisation/monitoring of progress, etc.). We have found that presenting content *via* brief (2–3 min) “consumer documentary videos” is a powerful engagement strategy [see Ref. (75)]. Other mature approaches to digital intervention elevate social network features for their engagement and therapeutic benefits [e.g., Refs. (72,)].

A major determinant of engagement is duration, and online therapy designers must juggle the desire for comprehensive topic coverage, attrition risk, and patients’ preference not to be hurried through content (9). The co-design process is critical for these decisions, with patients’ intuitions complemented by their participation in multiple prototype versions. For example, co-design of our intervention for late stage BD (54) led to a structure in which an initial “active phase” with email coaching support lasting 5 weeks; participants then retained access to the site (without coaching support) for the 6 months of follow-up. Based on our retention rates in that trial, we believe that a slightly longer active phase (e.g., the nine weekly content modules proposed in **Figure 2**) would be feasible and acceptable, but consumer input may modify this prediction.

Finally, intervention design must be sensitive to the fact that the Internet is now most commonly accessed by smartphone, with this device being particularly popular among young people (77, 78). Little is known about how engagement with online therapy content varies by device (79), but not only are smartphones the platform du jour, but they also offer engagement/intervention opportunities not available by website (think ecological momentary assessment and intervention, passive monitoring, etc.). There is reason to think these app-based technologies may be particularly impactful for engagement in young people (14).

### Minimizing Risk of and Responding to Clinical Events

The flexibility and reach of online interventions bring with them concerns about risk management (18, 80). We have reported on one successful approach to risk management in online intervention for late-stage BD. Our approach (again, strongly informed by consumer input) explicitly emphasizes patient autonomy and devolution of clinical care to local clinical services (18). A complexity in this strategy is that clinicians overseeing the online therapy may inadvertently come to know about increased risk, requiring some response: we have developed a decision-tree procedure (involving automated and manual components) to address this challenge (54).

Online intervention alone will be insufficient to optimize outcomes for some young people. Stepped care approaches are common in public health [e.g., Refs. (81, 82)], with progression from low- to high-intensity intervention triggered by deterioration/failure to improve. Stepped care has been considered for established BD [see Ref. (83)] and likely has particular relevance in the early intervention context. “Stepping up” for those not benefiting from digitally delivered psychotherapy could include intensive face-to-face psychotherapy and/or introduction of adjunctive pharmacotherapy (84).

### Generalization and Maintenance of Treatment Effects

A recognized challenge in psychotherapy delivery (whether face-to-face or digital) is generalization of new skills and insights into everyday life. The potential of smartphones to bridge this gap is significant, and a number of groups are investigating apps (either stand-alone or integrated with web-based modules) as part of mobile monitoring and therapy delivery (85–87). It would be unwise to ignore this technological trend in any future early intervention for BD.

Adopting the “active phase”/”follow-up phase” structure described in the section *Access and Engagement* previously, the follow-up phase, could include periodic booster modules to help maintain therapeutic benefits. Booster modules could provide 1 week of new content, with topics personalized for the individual (based on their preferences, symptoms, and problems remaining at the end of the active phase, etc.).

### Co-Design

Research by our group [e.g., Ref. (61)] confirms the common-sense intuition that participatory research methods (involving end-users in all stages of intervention development) not only circumvents translational barriers but helps accelerate novel treatments by testing theoretical and empirically derived ideas against lived experience (88). Here, the systematic co-design phase would complete pieces of work around therapeutic content (refining **Figure 2**) and trial and provide feedback on technology prototypes, participate in design of social networking components, etc.

## Conclusions

I have previously argued that the task of developing online interventions in mental health has little in common with developing face-to-face psychotherapy manuals: Engagement is the sine qua non of digital interventions, and designing an online intervention has much in common with producing a series for Netflix (89). The engagement challenge is particularly pointed in the case of digital natives at risk of, but not diagnosed with, BD. In this population, we cannot assume that “need” is a sufficient driver of engagement, and we must speak to the positive motivations that might lead people to stick with our therapeutic offer. The present perspective paper posits that these engagement-related considerations align neatly with theoretical considerations about early intervention in BD: People at elevated risk of BD must be viewed through a teleological lens that includes their developmental tasks and opportunities, their current psychological challenges, and their risk of future problems (including diagnosable BD). An evidence-informed intervention design framework is offered as a translational tool to support further work in this important domain.

## Author Contributions

GM conceived and wrote the article.

## Conflict of Interest Statement

The author declares that the research was conducted in the absence of any commercial or financial relationships that could be construed as a potential conflict of interest.

## Footnotes

1 For the present purposes, the phrase “early intervention” is preferred over “prevention” because a) from a staging perspective, people with elevated risk of a future diagnosis of BD (through, e.g., family history and nonspecific symptoms) can be viewed as being on the developmental spectrum of BD, and b) the goals of optimal intervention in this population are much broader than prevention of a future specific BD diagnosis.

2 I have previously highlighted the dispiriting and unwarranted (cancer-related) terminology currently used in BD staging research (28). As an alternative to 'stage', we have proposed the term, ‘experience with BD’(ranging from limited to substantial) which is both less dispiriting, and avoids unsupported connotations about the nature of BD's trajectory.

3 From a strong transdiagnostic perspective, it might be argued that the population of interest in this paper (young people at risk of BD) does not warrant delineation at all. However, existing literature provides grounds for a hybrid transdiagnostic/diagnostic approach, and we would be needlessly ignoring (relatively) BD-specific information (the importance of sleep and circadian stability, the ambiguity of elevated mood states, family history of BD diagnosis, etc.) if we adopted a fully diagnosis-blind approach.

4 The design of an early intervention for BD will depend on the nature and definition of the risk population to which it is offered. The clinical aim of, specifically, preventing transition to BD in high-risk young people has generated a small literature on quantifying this particular risk [see Refs. (50, 51)].

5 The co-design process could lead to refinement of the considerations presented here, and different co-design processes could lead to different testable interventions.

6 The most common trajectory in BD is one or more early depressive episodes, followed by an episode of hypomania or mania that then supports a BD diagnosis.
